# Biochemical and structural characterization of a mannose binding jacalin-related lectin with two-sugar binding sites from pineapple (*Ananas comosus*) stem

**DOI:** 10.1038/s41598-018-29439-x

**Published:** 2018-07-31

**Authors:** Mohamed Azarkan, Georges Feller, Julie Vandenameele, Raphaël Herman, Rachida El Mahyaoui, Eric Sauvage, Arnaud Vanden Broeck, André Matagne, Paulette Charlier, Frédéric Kerff

**Affiliations:** 1 Université Libre de Bruxelles, Faculty of Medicine, Protein Chemistry Unit, Campus Erasme (CP 609), 808 route de Lennik, 1070 Brussels, Belgium; 2 Laboratory of Biochemistry, Center for Protein Engineering-InBioS, Institute of Chemistry B6a, University of Liège, 4000 Liège, Belgium; 3 Laboratory of Enzymology and Protein Folding, Centre for Protein Engineering-InBioS, Institut de Chimie B6, University of Liège, 4000 Liège, Belgium; 4 Laboratory of crystallography, Center for Protein Engineering-InBioS, B5a, University of Liège, 4000 Liège, Belgium

## Abstract

A mannose binding jacalin-related lectin from *Ananas comosus* stem (AcmJRL) was purified and biochemically characterized. This lectin is homogeneous according to native, SDS-PAGE and N-terminal sequencing and the theoretical molecular mass was confirmed by ESI-Q-TOF-MS. AcmJRL was found homodimeric in solution by size-exclusion chromatography. Rat erythrocytes are agglutinated by AcmJRL while no agglutination activity is detected against rabbit and sheep erythrocytes. Hemagglutination activity was found more strongly inhibited by mannooligomannosides than by D-mannose. The carbohydrate-binding specificity of AcmJRL was determined in some detail by isothermal titration calorimetry. All sugars tested were found to bind with low affinity to AcmJRL, with *K*_*a*_ values in the mM range. In agreement with hemagglutination assays, the affinity increased from D-mannose to di-, tri- and penta-mannooligosaccharides. Moreover, the X-ray crystal structure of AcmJRL was obtained in an apo form as well as in complex with D-mannose and methyl-α-D-mannopyranoside, revealing two carbohydrate-binding sites per monomer similar to the banana lectin BanLec. The absence of a wall separating the two binding sites, the conformation of β7β8 loop and the hemagglutinating activity are reminiscent of the BanLec His84Thr mutant, which presents a strong anti-HIV activity in absence of mitogenic activity.

## Introduction

Lectins are carbohydrate-binding proteins widely distributed in nature. They have been reported in animals, insects, viruses, fungi and bacteria^1–3^, although the majority of them have been characterized from plants^4^. Lectins constitute a class of proteins/glycoproteins of nonimmune origin capable to bind reversibly and with a very high degree of specificity for mono- and oligosaccharides^5^. Lectins can trigger a number of biological effects through their interaction, e.g., with cell walls and membranes, including agglutination of erythrocytes^6,7^. Consequently, they were described as interesting tools in many research fields and biomedicine^8–12^.

In plants, based on careful analysis of the available protein sequences encoding lectins or lectin domains, sequence similarities and evolutionary relationships, twelve carbohydrate-binding domains (families) were classified^13^, including the so-called Jacalin-related lectin (JRL) family^14^. Structural studies on this family of lectins revealed a marked evolutionary plasticity within its members^15^. Though all members of this still expanding lectin family share high sequence similarity and are built up of protomers with a similar overall fold, they differ from each other regarding molecular structure of the protomers, their carbohydrate-binding specificities, post-translational processing and subcellular compartmentalization. Accordingly, the JRL are now subdivided into two subfamilies, the galactose- and mannose-specific JRL (gJRLs and mJRLs, respectively)^16^.

The galactose-specific JRLs constitute a small homogeneous group of galactose/T-antigen-binding lectins isolated exclusively from the Moraceae plant family. It was reported that jacalin from *Artocarpus integrifolia*, the first member identified in this family, follows the secretory pathway and accumulates in storage protein vacuoles^17^. The currently known galactose binding jacalin-related lectins closely resemble jacalin that can be considered as a prototype of this subfamily. The native gJRLs are built up of four identical protomers each consisting of a heavy (α) and a light (β) polypeptide chain. They undergo a complex processing mechanism of co- and post-translational proteolytic modifications from one pre-pro-protein to two separate chains linked together by noncovalent interactions^18^.

On the other hand, the mannose-specific jacalin-related lectins (mJRLs) constitute a growing group of lectins that occur in a wide variety of species belonging to different taxonomic groups, including monocots, dicots, cycads and ferns, and exhibiting carbohydrate specificity against mannose and oligomannosides^19–22^. In contrast to jacalin, native mJRLs are built up of two, four or eight protomers consisting of a single unprocessed polypeptide chain. They are synthesized without a signal peptide and are thus presumed cytoplasmic proteins^21,23^. They probably do not undergo co- or post-translational proteolytic modifications^24^. Interestingly, comparative structural and specificity studies carried out on the galactose binding lectin jacalin and the mannose binding homologues artocarpin and KM+ (from jackfruit), heltuba (from *Helianthus tuberosus*) and calsepa (from *Calystegia sepium*) showed that the differences in specificity between both subgroups rely on the presence or absence of the protomer proteolytic processing. In the absence of a proteolytic cleavage of the lectin polypeptide in the vicinity of the binding site, the mannose binding jacalin-related lectins retain an extra loop, which makes the binding site inaccessible to galactose. In contrast, when the protomer is cleaved into α- and β-chains, this extra loop is opened, extending thus the binding site, which preferentially binds galactose but also accommodates glucose, sialic acid and N-acetylmuramic acid^25^.

The widespread distribution of mannose-binding proteins among the plant kingdom put forward the high biological significance of the recognition/binding of mannose-containing glycans. Additional proof for the importance of the efficient mannose-recognition system is associated to the finding that higher plants have evolved to develop at least three different structural motifs to recognize/bind this saccharide, including the β-prism fold characterizing the mannose binding subgroup of the mJRLs^7,14,26^. It is interesting to note that while all known mJRLs share the basic ability to bind mannose⁄glucose, they clearly differ in fine specificity. X-ray crystallography and modeling studies carried out on different mJRLs homologues^23,27–32^ have indeed revealed that the protomers of all of these lectins adopt essentially the same β-prism fold, which is also common to Jacalin, the representative of gJRLs^33^. Interestingly, some adaptability of the β-prism fold as a building block was highlighted by the discovery of a novel circular arrangement^30^. Additionally, except for banana lectin (BanLec) and the lectin from *Cycas revoluta*^34^, the currently known mJRLs contained one sugar-binding site per protomer, which accommodates mannose and glucose as well as their derivatives. Structural analyses also revealed that key residues required for saccharides binding are essentially located on two loops, β1–β2 and β11–β12. These key residues were found highly conserved within the whole mJRLs family (or subfamilies), whereas other more divergent loops were described in the vicinity of these conserved residues. Therefore, the different specificities shown by mJRLs for complex glycans are expected to reflect the binding characteristic features of individual mJRLs^35^. Besides, an interesting data reported by Nakamura-Tsuruta *et al*.^36^ established that information on the sugar-binding specificities of mJRLs is more precisely revealed by their amino acid sequences than their biological phylogeny.

The exact physiological role fulfilled by lectins in plants, especially mJRLs, is not yet exactly known. However, given the rather scarcely distribution of mannose in the plant kingdom and its common presence on glycoconjugates structures exposed on the fungi, bacteria and viruses surfaces, it is tempting to suggest that mannose-binding lectins may play a defensive role against pathogenic microorganisms^7,37–39^. Interestingly, Bourne *et al*.^23^ described a mannose binding jacalin-related lectin from *Helianthus tuberosus* (heltuba) that accommodates Manα1–2Man and Manα1–3Man. These dimannosides are not common in plants, suggesting a defensive role of this lectin against foreign glycans.

In the present work, we present the purification and biochemical characterization of a mJRL from the stem of *Ananas comosus* (AcmJRL). We also determine its structure both in apo and complexed forms with D-mannose and methyl-α-D-mannopyranoside. AcmJRL constitutes, along with banana lectin (BanLec)^31^ and the lectin from *Cycas revoluta*^34^ the third member of mJRLs with two sugar-binding sites per protomer.

## Results and Discussion

### Purification of *A*. *comosus* lectin

**Figure 1:**
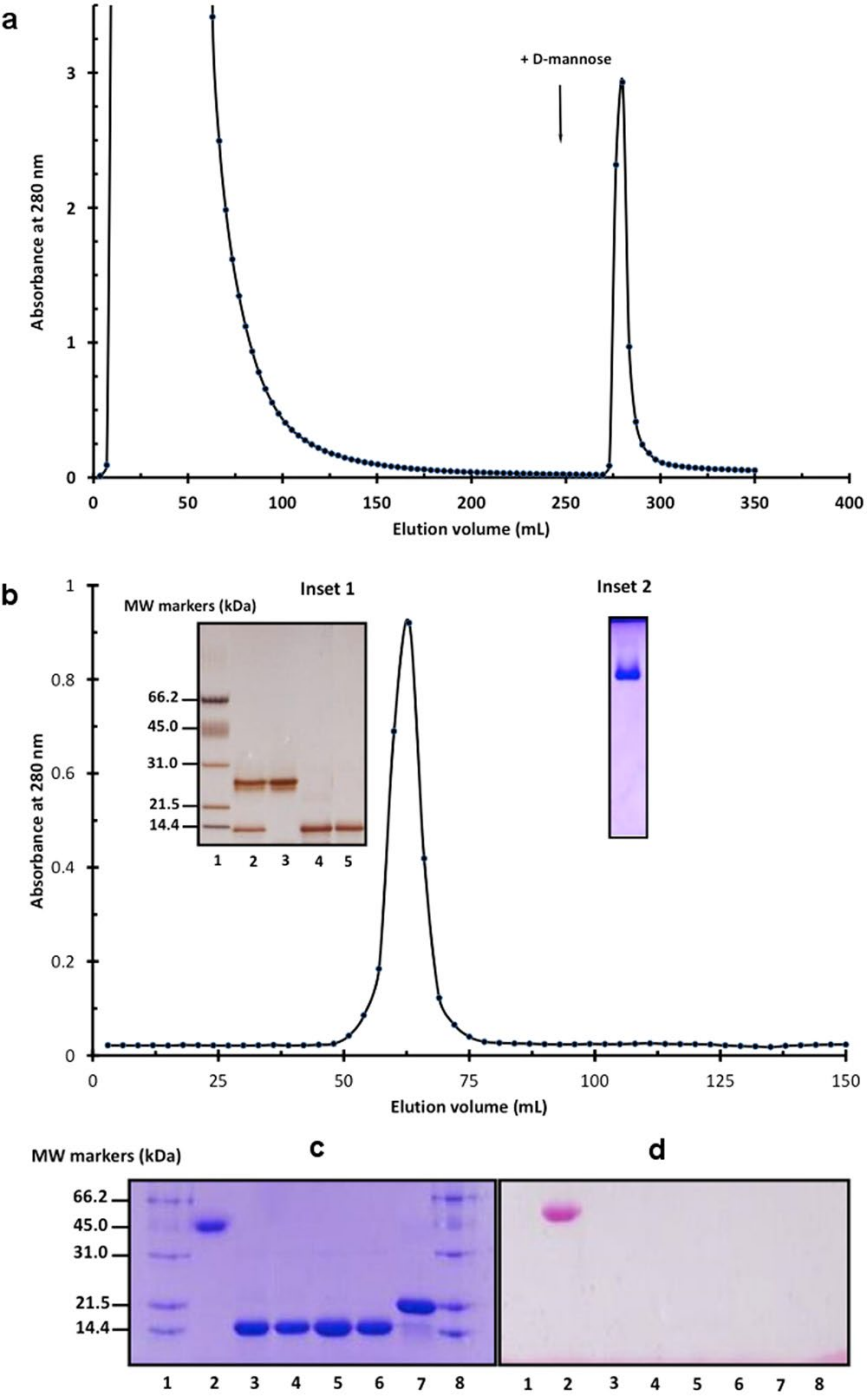
Purification and glycosylation state of AcmJRL. (**a**) Affinity chromatography of AcmJRL on D-mannose-Agarose, the arrow indicates the starting elution with D-mannose. (**b**) FPLC-gel filtration chromatography on Superdex 75 of the affinity-purified AcmJRL. Inset 1: SDS-PAGE. Lane 1: molecular weight standards, lane 2: total protein soluble fraction of *A*. *comosus* stem, lane 3: affinity chromatography flow-through fraction, lane 4: affinity chromatography retained fraction, lane 5: FPLC-gel filtration chromatography of the affinity chromatography retained fraction. Inset 2: native PAGE of AcmJRL after FPLC-gel filtration chromatography. (Glycan detection experiments on AcmJRL. (**c**) SDS–PAGE and (**d**) glycoprotein detection. 1 and 8: molecular weight standards, 2: positive control (horseradish peroxidase), 3–6: different preparations of AcmJRL, 7: negative control (soybean trypsin inhibitor).

As shown in Fig. 1a, the affinity column of D-mannose-Agarose separated the total protein soluble fraction from *A*. *comosus* stem into two fractions, a flow-through fraction and a retained fraction. The flow-through fraction contained mainly the cysteine proteases as revealed by their apparent molecular weight of 24 kDa (Fig. 1b, line 3 of Inset 1) and the protease activity measured after reduction of their catalytic cysteine residues by dithiolthreitol. The retained fraction was eluted by D-mannose and contained molecular species with apparent molecular weight of 14 kDa (Fig. 1b, lane 4 of Inset 1). N-terminal sequencing up to the 7^th^ amino acid (SGLVGLG) clearly identified the protein band as jacalin-like lectin from *A*. *comosus* (SwissProt entry: Q53J09_ANACO), although the N-terminal methionine residue documented in the SwissProt sequence was not detected. To further improve its purity, the retained fraction was subjected to FPLC-gel filtration chromatography. The sharp and symmetrical peak obtained after this step (Fig. 1b) suggested a homogenous protein preparation. In agreement, both native (Fig. 1b, Inset 2) and SDS–PAGE experiments (Fig. 1b, lane 5 of Inset 1) reflected the high purity and homogeneity of *A*. *comosus* lectin. N-terminal sequencing also argued for protein homogeneity as only one sequence was obtained. Besides, accurate molecular mass determination by ESI-Q-TOF-MS revealed one major peak of 15389.0 ± 1.5 Da (Supplementary Fig. 1). This value should be compared with *Mr* = 15346.4 Da deduced from the amino acid sequence (Met removed). The experimental molecular mass obtained by mass spectrometry is therefore likely the result of a di-sodium adduct of this form of AcmJRL (15346 Da + 2 × 23 Da = 15392).

Lectins are known to undergo oligomerization in solution. The molecular mass of native *A*. *comosus* mannose jacalin-related lectin (AcmJRL), as estimated using FPLC-gel filtration chromatography, was ~30 kDa, suggesting that AcmJRL was a homodimer in solution. To check if the AcmJRL was a glycoprotein, it was subjected to carbohydrate analysis by periodic acid oxidation. The results, shown in lines 3–6 of Fig. 1c,d, clearly demonstrated that AcmJRL was not glycosylated, as also indicated by the experimental mass determination. AcmJRL resembles thus other mannose specific jacalin-related lectins that were not glycosylated, such as artocarpin^40^ and heltuba^41^, while the galactose specific lectin jacalin^40^ was glycosylated.

### Hemagglutinating activity

Hemagglutinating assays revealed that AcmJRL showed no hemagglutination activity of rabbit and sheep erythrocytes, in the absence or presence of 50 mM of the divalent ions Mg^2+^, Mn^2+^ Zn^2+^ and Ca^2+^. In contrast, AcmJRL readily hemagglutinates rat erythrocytes (Supplementary Fig. 2) with a specific hemagglutinating activity of 62.5 µg/mL, a value higher compared with that (8.9 µg/mL) of the lectin purified from Chinese leek seeds^42^ and the mannose specific lectin purified from mulberry seeds^43^. Other mannose binding lectins were reported to be unable to hemagglutinate sheep erythrocytes^44,45^, but the striking difference in the hemagglutinating activity of AcmJRL compared to the majority of mannose binding lectins described to date is the inability of AcmJRL to hemagglutinate rabbit erythrocytes^21,41,46,47^. However, comparing agglutination activity of lectins between laboratories has to be taken with caution, as the results can be highly dependent on the erythrocyte preparation and experimental protocol.

Lectin oligomerization was described as a possible factor enhancing both affinity and selectivity by generating multiple sites for carbohydrate binding^48,49^. Banerjee *et al*.^50^ reported a homodimeric lectin from *Allium sativum*, ASAL, which loses its hemagglutinating property when engineered to a stable monomeric form of the lectin, mASAL. These authors suggested that the loss of the hemagglutination activity results from a smaller number of carbohydrate binding sites in the monomeric lectin form that consequently causes a loss of the multivalency essential for hemagglutination. Size-exclusion chromatography clearly showed that AcmJRL is a homodimeric lectin in solution. The absence of hemagglutinating activity of rabbit erythrocytes cannot thus be explained by the absence of oligomerization. Furthermore, many mannose specific lectins displaying hemagglutination activity against rabbit erythrocytes are described as dimeric in solution^21,41,45,46,51^.

The results of hemagglutination inhibition assays of AcmJRL (62.5 µg/mL final concentration) indicated that the mannopentaose αMan(1,3) (αMan(1,6))-αMan(1,6) (αMan(1,3))Man exerted the highest inhibitory effect, with a MIC value of 0.32 mM followed by the mannotriose αMan(1,3) (αMan(1,6))Man (0.47 mM) and by the dimannose Man-α-1,3-Man (0.94 mM). Furthermore, minimum inhibitory concentration required for both methyl-α-D-mannopyranoside and methyl-α-D-glucopyranoside was 1.56 mM while for D-glucose and GlcNAc the MIC value was 3.12 mM. D-mannose presented the lowest inhibitory effect, with a MIC value of 6.25 mM. These data (summarized in Supplementary Table 1) demonstrate that AcmJRL displays preference for oligosaccharides compared to monosaccharides. Other mannose specific lectins exhibited similar behavior. These are, e.g., the lectins from *Phlebodium aureum* rhizomes and *Centrolobium microchaete* seeds and heltuba^19,47,52^.

### Equilibrium unfolding experiments

**Figure 2:**
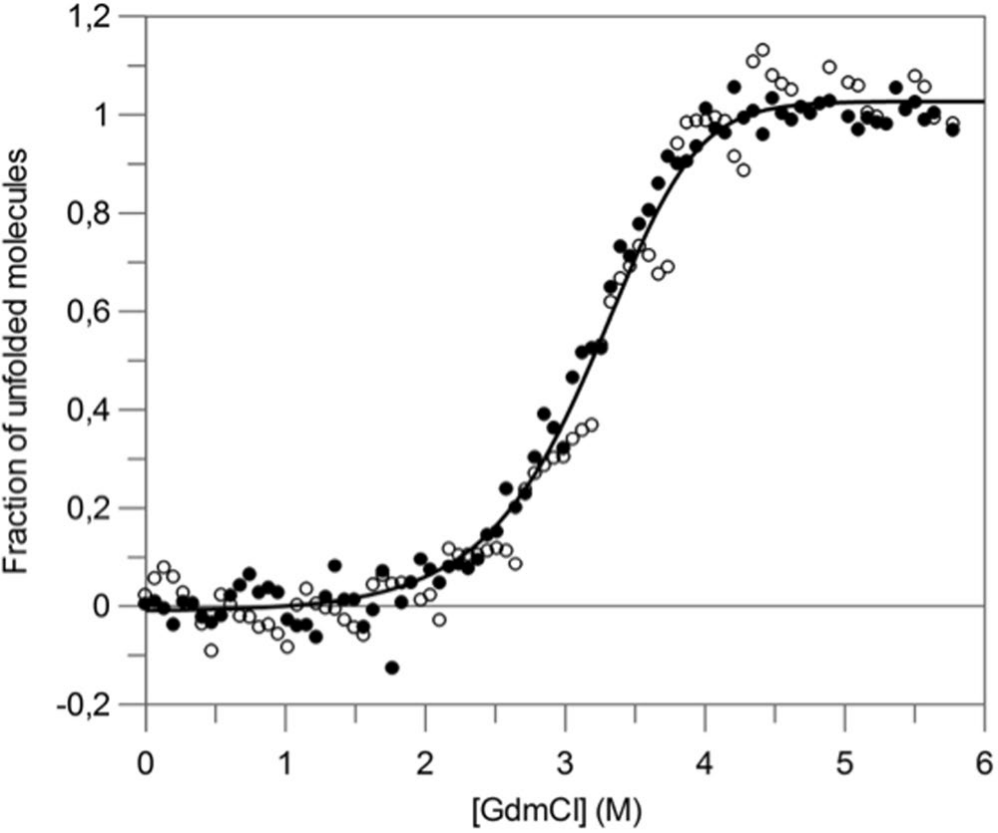
GdmCl-induced equilibrium unfolding transition of AcmJRL at pH 7.0 and 20 °C, monitored by the change in both fluorescence intensity at 370 nm (•) and in ellipticity at 222 nm (○). Data were analyzed on the basis of a two-state model (N_2_ ⇌ 2U)^71^, and the solid line was drawn using the mean values of the parameters obtained by both techniques (Table 1). Data are presented as the fractional change in signal as a function of GdmCl concentration.

The coincidence of the curves (Fig. 2) obtained by intrinsic fluorescence and Far-UV CD measurements, in the presence of various concentrations of guanidinium chloride (GdmCl), indicates that secondary and tertiary structures are destabilized simultaneously. These results suggest that the protein unfolds in an apparent two-state transition (N_2_ ⇌ 2U), where only the native dimer and the unfolded monomer are significantly populated. Analysis of the data in Fig. 2, according to a two-state model for a homodimeric protein, allowed computation of the characteristic thermodynamic parameters, i.e. (ΔG°(H_2_O)_NU_ = 59.1 ± 0.1 kJ·mol^−1^) and m_NU_ = −9.4 ± 0.3 kJ·mol^−1^·M^−1^ (for further details, see Table 1). Similar results were obtained by Gupta *et al*.^53^ for the unfolding of banana lectin, which proved to be *ca* 3 kcal·mol^−1^ more stable than AcmJRL.

**Table 1:** Thermodynamic parameters of unfolding of AcmJRL at pH 7 and 20 °C, as obtained from analysis of equilibrium transitions monitored by both intrinsic fluorescence and Far-UV CD measurements.

Technique	Δ*G*°(H_2_O)_NU_ (kJ·mol^−1^)	−*m*_NU_ (kJ·mol^−1^·M^−1^)	*C*_m_ (M)
Intrinsic fluorescence	59 ± 4	9.2 ± 1.1	3.3 ± 0.4
Far-UV CD	59 ± 2	9.6 ± 0.7	3.1 ± 0.3

### Isothermal titration calorimetry (ITC)

**Figure 3:**
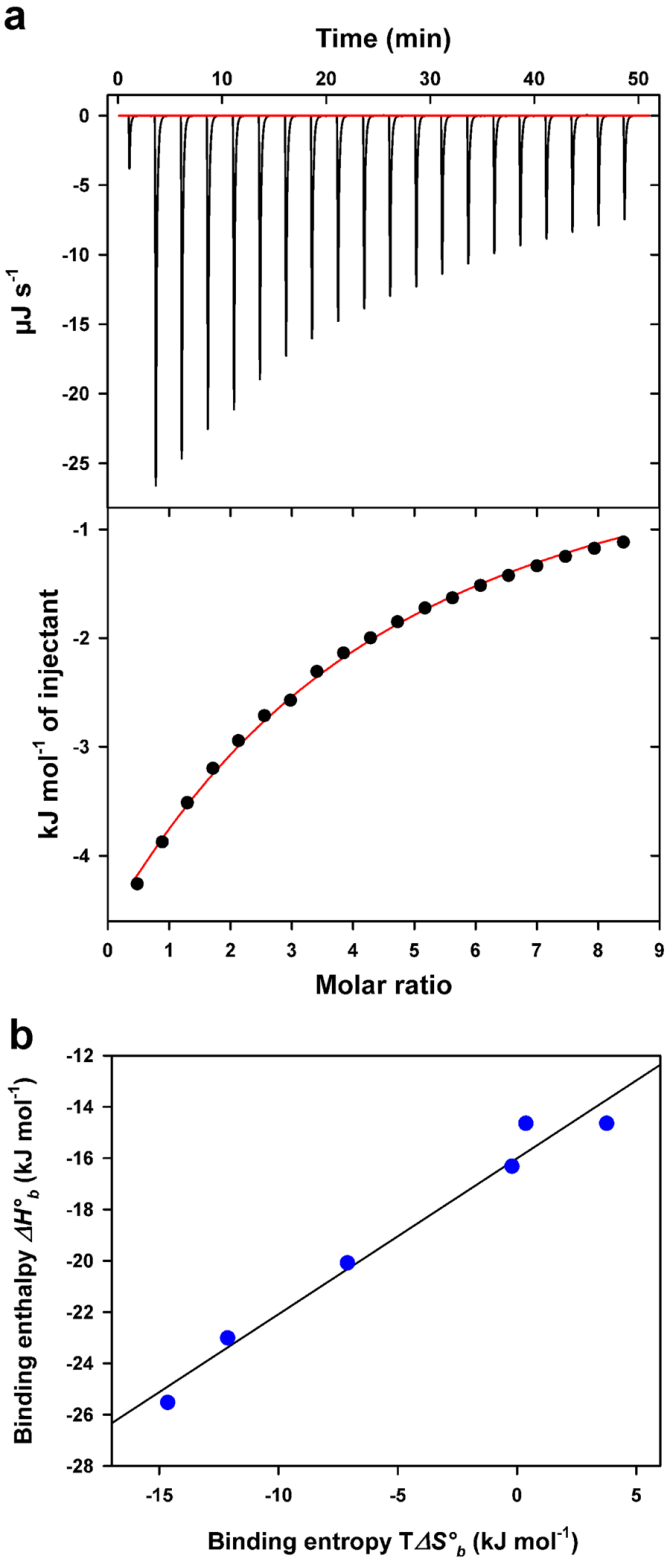
Isothermal titration calorimetry of D-mannose binding to AcmJRL and Enthalpy-entropy compensation plot. (**a**) Upper panel: exothermic microcalorimetric traces of D-mannose (32 mM) injections into AcmJRL solution (0.8 mM). Lower panel: Wiseman plot of heat releases versus molar ratio of injectant/protein in the cell and nonlinear fit of the binding isotherm for 2 equivalent binding sites. (**b**) Enthalpy-entropy compensation plot of sugar binding to AcmJRL. Enthalpy and entropy values are from Table 2. The plot displays a linear relationship with *r*^2^ = 0.97.

In order to study the interactions of AcmJRL with potential ligands, the lectin was titrated with mono- and oligosaccharides, using ITC (Supplementary Fig. 3). This system records the heat generated by association of the binder with its ligand and following progressive saturation, the binding enthalpy, the affinity constant and the stoichiometry are derived from a Wiseman plot (Fig. 3a, lower panel). The various sugars used here were selected from those already investigated for the mannose binding jacalin-related lectin artocarpin^27,54^. All sugars tested were found to bind with low affinity to AcmJRL, with dissociation constants in the mM range. For such low affinity systems, full saturation by the ligand cannot be reached because of technical limitations. Accordingly, only a partial binding isotherm is recorded (Fig. 3a) and one of the fitted variables has to be kept constant, usually the binding stoichiometry^55^. In the present case, on the basis of two mannose molecules bound to the X-ray structure of AcmJRL, the thermodynamic parameters of sugar binding were calculated for a fixed stoichiometry *n* = 2. Furthermore, the fitted binding enthalpy *ΔH°*_*b*_ was checked independently by direct injections of AcmJRL aliquots into a saturating concentration of saccharide (20 × Kd) as described^56^ (Supplementary Fig. 4). Both fitted and experimental enthalpies were found to agree within 5–10%.

AcmJRL does not bind galactose and it is therefore not related to galactose-binding lectins. In contrast, as shown in Table 2, the association constant *K*_*a*_ for the various sugars follows the same trend than that reported for artocarpin^57^, with a weak affinity for monosaccharides and increasing affinity from mannose to di-, tri- and penta-mannooligosaccharides. Data from Table 2 showed that AcmJRL binds to the mannopentaose with a 10-fold higher affinity compared to mannose. The same behavior was observed with the hemagglutination inhibition assays. Interestingly, AcmJRL differs by its ability to bind GlcNAc, in contrast to artocarpin. However, the *K*_*a*_ values for AcmJRL are one order of magnitude lower than those reported for artocarpin and suggest that the tested sugars are potentially not the physiological ligands of AcmJRL. The large fitting errors on *K*_*a*_ for mannooligosaccharides may also indicate that both observed binding sites in the crystal structure are not fully equivalent for sugar binding. Fitting the ITC data with *n* = 1 reduces the error on *K*_*a*_ but provides binding enthalpy values much higher than those reported for lectins (Supplementary Table 2). This again indicates that two sites contribute to saccharide binding, although differently. Bridging between the two binding sites by a branched glycan has been recently reported for JRLs^58,59^. A similar bridging in AcmJRL with mannopentaose will be discussed thanks to the structures presented below. ITC data cannot discriminate such binding mode. The enthalpic and entropic contributions to *ΔG°*_*b*_ show that the association is enthalpy-driven by favorable interactions such as H-bonds or van der Waals contacts^60^. Furthermore, these parameters exhibit strong enthalpy-entropy compensation (Fig. 3b) characterized by a linear relationship. Such behavior primarily indicates that sugar recognition and binding by AcmJRL occur via the same mechanism for the tested saccharides. The compensation phenomenon is expected to originate from increased bonding (more negative *ΔH°*_*b*_) at the expense of increased order (more negative T*ΔS°*_*b*_ as shown by monosaccharides), whereas solvent reorganization at the binding surfaces tend to render the entropic term more favorable, as shown for mannooligosaccharides^60^. The majority of mannose binding jacalin-related lectins has been described to occur as dimeric and tetrameric forms in solution^15,38,41,61^, though a monomeric form was also reported^62^. This diversity in quaternary structures highlights the important role of these oligomeric assemblies for carbohydrate recognition events, both in term of affinity and specificity^29,48^. Interestingly AcmJRL showed more comparable but still lower saccharide binding affinity when compared to two mJRLs from banana (BanLec and plantain) that were also described as dimeric forms in solution^63^.

**Table 2:** Thermodynamic parameters of saccharide binding to AcmJRL at 25 °C for 2 equivalent binding sites (n = 2).

Saccharide	*K*_*a*_ (M^−1^)	*ΔG°*_*b*_ (kJ mol^−1^)	*ΔH°*_*b*_ (kJ mol^−1^)	T*ΔS°*_*b*_ (kJ mol^−1^)
D-Glucose	83 ± 3	−11.0 ± 0.4	−23.3 ± 0.6	−12.3
GlcNAc	88 ± 1	−11.1 ± 0.1	−25.6 ± 0.2	−15.5
D-Mannose	178 ± 4	−12.8 ± 0.3	−20.0 ± 0.3	−7.2
Manα1,3Man	421 ± 52	−15.0 ± 1.8	−14.6 ± 1.0	0.4
Mannotriose^(a)^	734 ± 117	−16.3 ± 2.6	−16.5 ± 1.2	−0.2
Mannopentaose^(b)^	1694 ± 679	−18.4 ± 6.7	−14.5 ± 2.0	3.9

^(a)^αMan(1,3)(αMan(1,6))Man.

^(b)^αMan(1,3) (αMan(1,6))-αMan(1,6) (αMan(1,3))Man.

### Overall structure of AcmJRL

The crystal structure of AcmJRL was determined to 1.8 Å resolution using as search model the banana lectin (pdb code 1 × 1 V)^31^, the structure from the Protein Data Bank with the highest sequence identity (42%). This structure belongs to the P2_1_2_1_2_1_ space group and is characterized by R_work_ and R_free_ of 14.6% and 18.3% respectively (see Table 3 for additional model refinement statistics). The asymmetric unit contains four monomers. Each monomer starts at Ser2 and ends at Tyr145, with a well-defined carboxy terminal group, in agreement with the sequence reported in the UniProtKB database (Q53J09_ANACO) except for the absence of the N-terminal methionine also not detected by N-terminal sequencing.

**Table 3:** Crystallographic data and model refinement statistics.

	AcmJRL	AcmJRL:Man	AcmJRL:MeαMan
**Data Collection:**			
Wavelength (Å)	1.214580	0.9785	0.9785
Space group	*P2*_1_2_1_2_1_	*P6*_4_22	*P6*_4_22
a, b, c (Å)	68.1, 86.0, 88.9	86.7, 86.7, 257.3	85.9, 85.9, 204.4
α, β, γ (°)	90, 90, 90	90, 90, 120	90, 90, 120
Resolution range (Å)^a^	45.8–1.8 (1.9–1.8)	48.8–2.75 (2.92–2.75)	42.9–1.9 (2.0–1.9)
Rmerge (%)^a^	10.3 (81.2)	19.6 (127)	9.0 (92.8)
<I>/<σI>^a^	15 (2.7)	13.2 (2.0)	19.9 (2.9)
Completeness (%)^a^	100 (100)	99.9 (99.8)	99.7 (98.2)
Redundancy^a^	7.4 (7.3)	12.4 (12.1)	9.5 (9.4)
**Refinement:**			
Resolution range (Å)	44.6–1.8	48.8–2.75	42.9–1.9
No. of unique reflections	49023	28132	66445
R work (%)	14.6	18.7	15.8
R free (%)	18.3	22.1	17.2
No. atoms			
Protein	4348	2174	2174
Ligands	26	48	78
Water	502	58	271
RMS deviations from ideal stereochemistry			
Bond lengths (Å)	0.005	0.004	0.005
Bond angles (°)	0.8	0.98	0.8
Mean B factor (Å^2^)			
Protein	21.6	54.2	32.3
Ligands	46.8	72.2	62.9
Water	30.3	42.8	42.7
Ramachandran plot:			
Favoured region (%)	98.4	97.9	97.6
Allowed regions (%)	1.0	1.4	1.7
Outlier regions (%)	0.5	0.7	0.7

^a^Values in parenthesis refer to the highest resolution shell).

**Figure 4:**
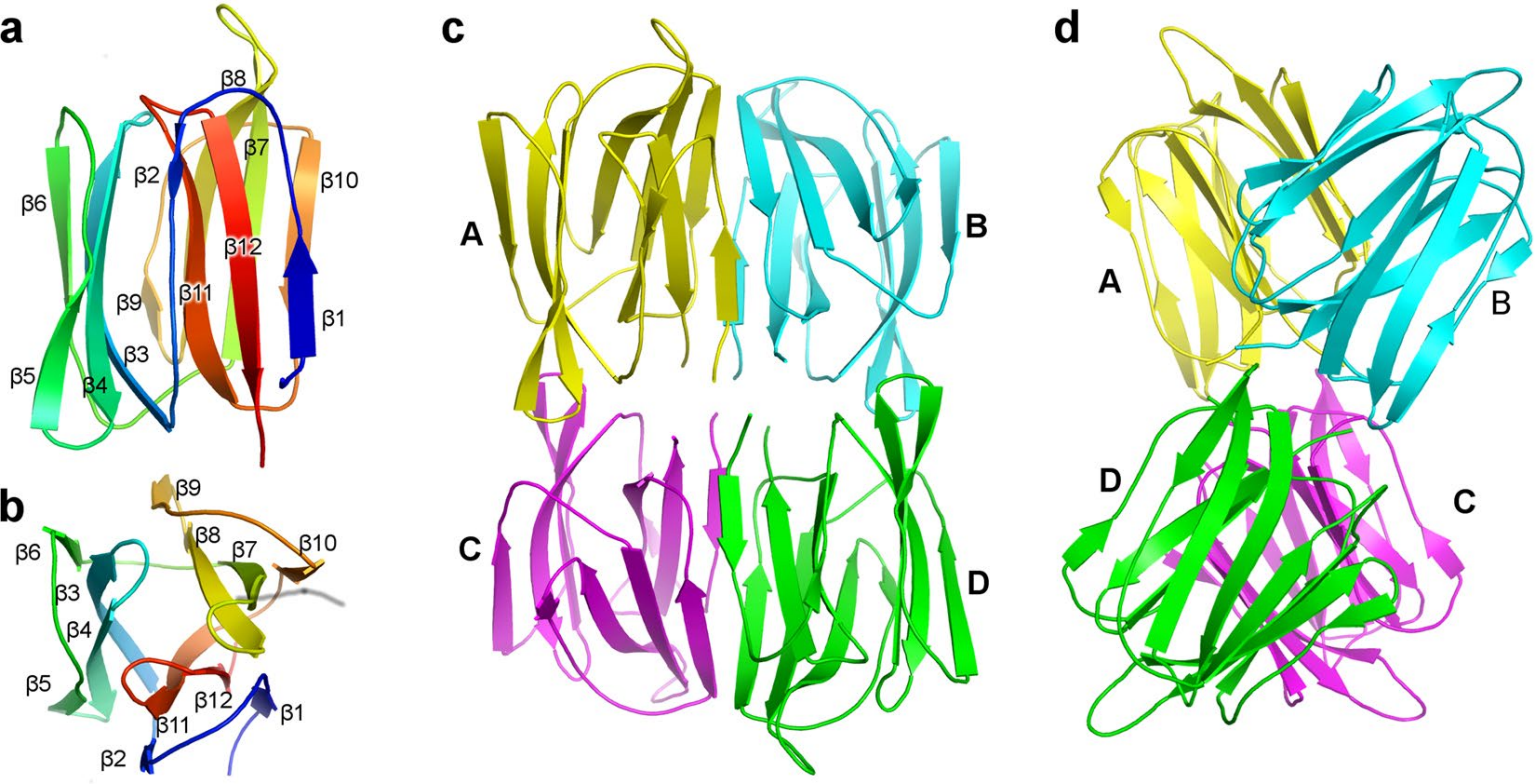
Overall fold of AcmJRL. (**a**) One monomer of AcmJRL is shown in cartoon representation with rainbow coloring from the blue N-terminus to the red C-terminus. The twelve β-strands are numbered. (**b**) Same as (**a**) with a 90° rotation around the horizontal axis. The three Greek key (β1–2 + β11–12; β3–6; β7–10) composing this fold are clearly separated. (**c**) Cartoon representation of the AcmJRL tetramer present in the crystal. (**d**) Same as (**c**) with a 90° rotation around the vertical axis.

AcmJRL adopt the expected β-prism fold (Fig. 4a,b) with the three characteristic Greek key four-stranded β-sheets (β1–2 + β11–12, β3–6 and β7–10) and all the connecting loops well defined in the electron density. Two densities were identified and attributed to citrate molecules. One is located in the carbohydrate-binding site 2 of monomer D and the second interacts with the β7β8 and β9β10 loops (Greek key 3) of monomer B.

**Figure 5:**
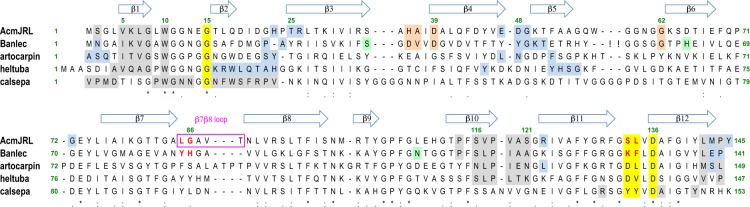
Multiple sequence alignment of relevant mJRLs. The alignment of AcmJRL, Banlec, artocarpin, heltuba and calsepa was obtained using Clustal W and improved thanks to the superimposition of the corresponding structures. Arrows above the alignment represent the β-strands of AcmJRL. The residues involved in D-mannose binding in site 1 and 2 are highlighted in yellow and orange, respectively. Residues binding the 3^rd^ D-mannose in Banlec are highlighted in green. Residues involved in side-by-side dimerization and higher order oligomerization (octamer for heltuba and tetramer for others) are highlighted in grey and blue respectively. The four residues forming the “wall” between the binding sites of AcmJRL and Banlec are showed in red.

The most likely biological assembly identified by the PDBePISA server (http://www.ebi.ac.uk/pdbe/pisa/) corresponds to the four monomers present in the asymmetric unit. Monomers A and C form a tail-to-tail interaction similar to monomers B and D and the AC and BD dimers are aligned side-by-side with an approximate angle of 45° (Fig. 4c and d). The strongest interactions are between monomers A and B, and C and D; they mostly involve strands β1 and β10 (Fig. 5) with an average buried surface of 826 Å^2^ and ΔG of −11.5 kcal/mol as calculated by PISA. On the other hand the A-C and B-D interactions have an average buried surface of 695 Å^2^ and ΔG of about −6.2 kcal/mol. This tetrameric form of AcmJRL differs from the dimeric form observed experimentally by size exclusion chromatography. The main cause of this discrepancy is likely the high protein concentration used for crystallization (1.1 mM) compared to size exclusion chromatography (0.15 mM) experiments. The same feature was observed for BanLec, which forms dimers in solution but is also found as a tetramer in the crystal structure^31^.

Despite this similarity, the quaternary structures of AcmJRL and BanLec are only partially similar. The side-by-side interaction involving strands β1 and β10 is indeed conserved with some amino acids substitution (V5I, L7V, V117L, V119L and S121A) approximately keeping the size and hydrophobic characteristic that drives this interaction (Fig. 5). The tail-to-tail interaction is however not conserved and generates a different tetramer than the one observed in BanLec. The same is also true for artocarpin, jacalin and the mJRLs from *Morus nigra* and *Maclura prolifera*^27,33,64,65^, who all share the same side-by-side dimerization as AcmJRL, but have a common tail-to-tail interaction different than AcmJRL and BanLec. The mJRL from *Helianthus tuberosus* (heltuba)^23^ forms donuts shaped octamers made of four basic dimers similar to AcmJRL, and the mJRL from *Calystegia sepium* (calsepa)^66^ only forms dimers different than the AcmJRL ones (Fig. 5). Finally, for the mJRL from *Ipomoea batatas* (Ipomoelin), the dimer is similar to calsepa, but the tetramer is stabilized by the swapping of a 10 amino acids N-terminal extension^67^.

### Saccharide binding sites

Two structures of AcmJRL in complex with D-mannose (AcmJRL:Man) and methyl-α-D-mannopyranoside (AcmJRL:MeαMan) were obtained at 2.75 Å and 1.9 Å respectively. These structures belong to the P6_4_22 space group but with significantly different c axes (257.3 Å and 204.4 Å respectively) indicating different packing. In both cases, the asymmetric unit contains two molecules. They form the side-by-side dimer in AcmJRL:Man and the tail-to-tail dimer in AcmJRL:MeαMan. In either case, the tetramer observed in the AcmJRL structure can be reconstructed using symmetry mates, the interactions between tetramers are different in the two crystals. The overall structures of the AcmJRL:Man and AcmJRL:MeαMan complexes are similar to the AcmJrl structure, with rms deviation between carbon α of the four monomers of 0.75 Å and 0.7 Å respectively.

**Figure 6:**
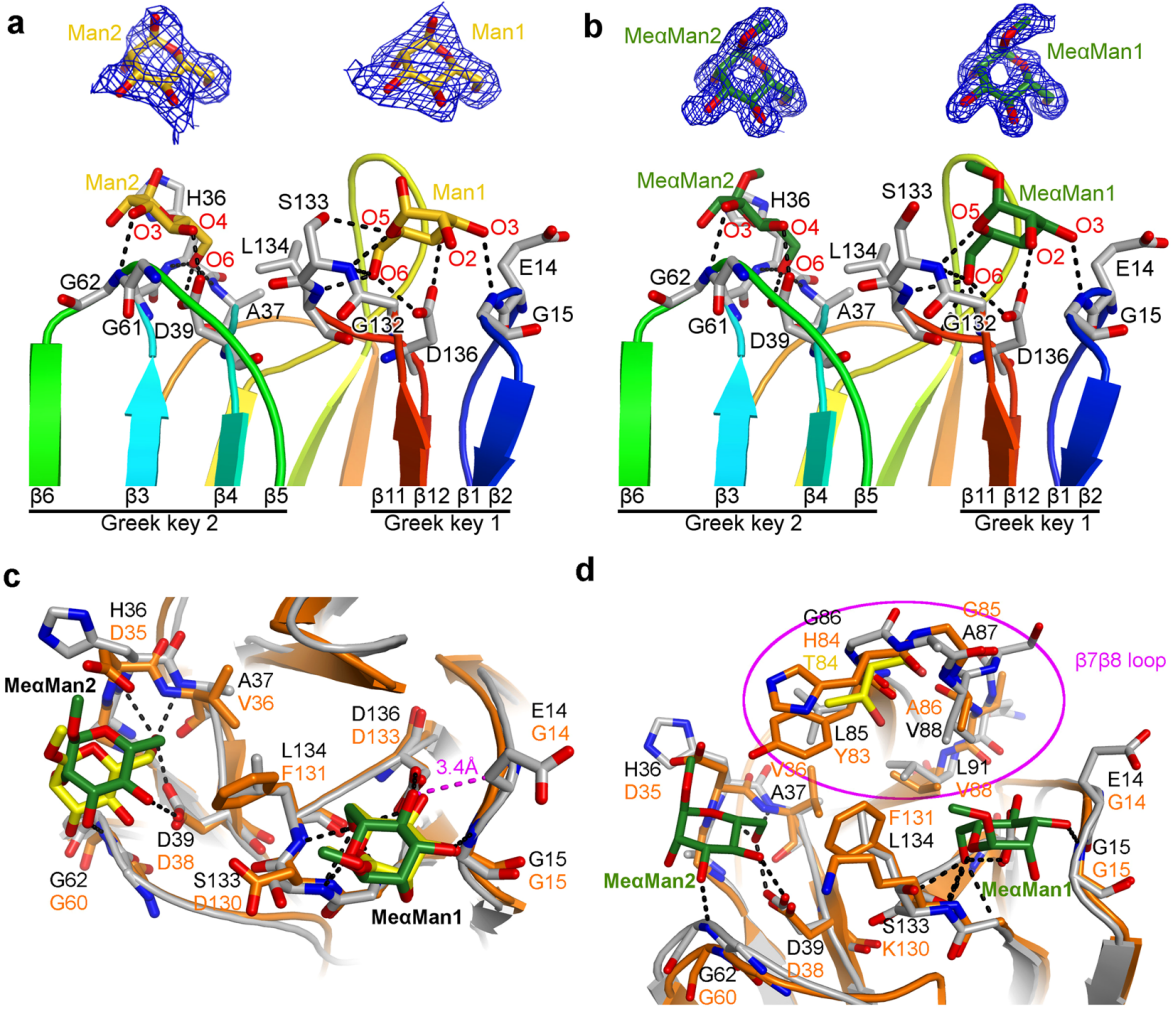
Interactions of the α-D-mannose and methyl-α-D-mannopyranoside in the carbohydrate-binding sites of AcmJRL and comparison with BanLec. (**a**) Monomer A of the AcmJRL:Man complex is shown in cartoon representation with rainbow coloring from the blue N-terminus to the red C-terminus. The D-mannose molecules are shown as yellow sticks, and the amino acids involved in their binding as grey sticks. The D-mannose binding site 1 and 2 are formed by the loops of the Greek key 1 and 2 respectively. Two inserts highlight the electron density of the feature-enhanced map at the 1σ level around the two D-mannose molecules. (**b**) Same as A for the AcmJRL:MeαMan complex, the methyl-mannose molecules being displayed in green. (**c**) Superposition of AcmJRL and BanLec in complex with methyl-α-D-mannopyranoside. AcmJRL is represented in grey cartoon and sticks and BanLec in orange. MeαMan molecules in complex with AcmJRL and BanLec are shown as green and yellow sticks respectively. The close contact between MeαMan1 and Glu14 in AcmJRL is highlighted in magenta. (**d**) Comparison of the β7β8 loops of AcmJRL and BanLec with the same representation style as (**c**). Thr84 of the BanLec His84Thr mutant is shown in yellow sticks.

In the AcmJRL:Man complex, four extra densities were interpreted as D-mannose molecules, two at the same positions in each monomer. One interacts with Greek key 1 (site 1) and one with Greek key 2 (site 2) (Figs 5 and 6a). In site 1, D-mannose oxygens are within hydrogen binding distance of the two carboxyl oxygens of Asp136 (O4 and O6), the hydroxyl of Ser133 (O5), and the backbone nitrogen of Gly15 (O3), Ser133 (O5) and Leu134 (O6). In site 2, the binding mode is similar to site 1, and the D-mannose oxygens interact similarly with the two carboxyl oxygens of Asp39 (O4 and O6) and the backbone nitrogen of Gly62 (O3), His36 (O5) and Ala37 (O6).

In the AcmJRL:MeαMan complex, a methyl-α-D-mannopyranoside molecule was modeled in each of the two binding-sites 1. The interactions with AcmJRL were all conserved except the hydrogen bond between O5 and the hydroxyl group of Ser133, which rotated 120° as a result of steric hindrance with the methyl group of this ligand (Fig. 6b). For the binding site 2, a methyl-α-D-mannopyranoside molecule was only modeled in monomer A. The hydrogen bonds between the ligands and the protein in the AcmJRL:Man complex where conserved in the AcmJRL:MeαMan complex, despite a slight rotation of the ligand potentially due to the close proximity of the ligand methyl and the side chain of His36. An extra density is also present in the binding site 2 of monomer B but the quality was not sufficient to properly fit the ligand. This is likely a consequence of an insufficient occupation of this site due to a steric hindrance with the methyl-α-D-mannopyranoside bound in the binding site 1 of a symmetric molecule in the crystal.

### Comparison of binding sites of the different mJRLs

The structure of AcmJRL is the second structure of a mJRL reported with two carbohydrate-binding sites per monomer after BanLec. The binding mode of D-mannose and methyl-α-D-mannopyranoside in the site 1 is similar to the one observed in the other structures of complexes between mJRLs (BanLec, artocarpin, *Morus nigra* mJRL and heltuba) and D-mannose or mannose-containing oligosaccharides with only subtle shifts of the β1–2 and β11–12 loops, which contribute to the binding^23,27,64,68^. In the case of the mannose-binding site 2 (Fig. 6c), the binding mode is also similar to the one observed in BanLec, but the shift in the β3–4 and β5–6 loops is slightly more pronounced (about 1.2 Å). In some BanLec structures (pdb codes 3MIU and 2BMY^31,68^), a third saccharide is detected close to Ser33, Asn106 and His63 (between Greek key 2 and 3). This binding site has not been discussed in the related publications and is not conserved in AcmJRL.

The affinity of AcmJRL for D-glucose and D-mannose is lower than the one reported for artocarpin and BanLec, despite a similar binding mode (same hydrogen bond network). However, except for the central aspartate, the atoms involved in these bonds are backbone nitrogens and variations in the identity of the side chains in the different mJRLs could affect the binding. Indeed, AcmJRL is characterized by a glutamate at position 14 in binding site 1 instead of a glycine in all other structures. In the AcmJRL:Man and AcmJRL:MeαMan complexes, the distance between the oxygen O4 of the ligand and Cβ of Glu14 is only 3.4 Å (Fig. 6c). This represents an unfavorable contact that could be responsible for the slightly lower affinity of AcmJRL for D-mannose and mannose-containing oligosaccharides. This glutamate is also likely to interfere with the binding in the site 1 of oligosaccharides containing a β1,3 link as observed in the structures of BanLec in complex with laminaribose and Xyl-β1,3-Man-α-O-methyl^31^. In binding site 2, no interference would however occur, making it difficult to evaluate the effect on the overall affinity of AcmJRL to reducing 3-O-β-linked sugar molecules. The physiological implication of this substitution remains also to be determined.

### Bridging between the two binding sites

Bridging between the two binding sites by a branched glycan has been reported for Banlec and other lectins^58,59^. Because of the similarity with Banlec, AcmJRL is expected to allow a similar bridging between its two binding sites. Mannopentaose is the largest oligosaccharide used in this study; it is made of three branches, each one being terminated by a D-mannose linked by its C1 carbon. A maximum of two mannose units are present in between these terminal saccharides, equivalent to a distance of maximum 10.5 Å between the oxygen atoms of the terminal links. In the complex structures of AcmJRL, the distance between the O1 oxygens of the two binding sites is about 14 Å, making it impossible for mannopentaose to be bound in both site in the conformations observed in the AcmJRL:Man complex structures. However, we cannot rule out that one mannose binds one site in the canonical conformation and a second terminal mannose interacts with the second binding site in a different conformation with lower affinity but still contributing to the overall binding and preventing the binding of a second molecule.

### Potential therapeutic use of AcmJRL as antiviral similar to BanLec?

BanLec has been shown to be a potent T cell mitogen and to avidly associate with high-mannose-type N-glycans on the envelope of HIV-1, hepatitis C virus, and influenza virus, thus blocking viral entry into cells^38^. Swanson *et al*.^69^ were able to engineer a single amino acid mutation (His84Thr) that maintains the antiviral activity of BanLec while dramatically reducing its mitogenic activity, which constitutes a potential side effect preventing potential clinical uses of lectins. This mutation is localized in the β7β8 loop (amino acids 83 to 86) of the third Greek key and is adjacent to both carbohydrate-binding sites (Fig. 6d). The effect of this mutation was linked to the disruption of a wall separating the two binding sites, reducing multivalent interactions that drive the mitogenic activity, while keeping a loop conformation appropriate for the high binding affinity for numerous viruses.

We found that AcmJRL is closely related to BanLec. It has 42% amino acid sequence identity and is a rare mJRL with two very similar carbohydrate-binding sites. In AcmJRL, the β7β8 loop maintains a conformation very similar to the BanLec one (Fig. 6d) despite being one amino acid longer and having no sequence identity (^85^LGAVTNL^91^ in AcmJRL vs ^83^YHGAVV^88^ in BanLec). In BanLec, the wall between carbohydrate binding sites is mainly constituted of four residues (Tyr83, His84, Lys130 and Phe131) that are all replaced by shorter residues in AcmJRL (Leu85, Gly86, Ser133 and Leu134) (Fig. 6d). In BanLec, the His84Thr mutation induces a size reduction of this wall that is correlated with an increase of the minimal concentration required for rabbit erythrocytes hemagglutination (from 3 to 437 μg/mL) and a decrease of the mitogenic activity. In AcmJRL, the more pronounced reduction of this wall is concomitant with an absence of detection of rabbit erythrocytes hemagglutination up to a concentration of 500 μg/mL, while the affinity for D-mannose is only slightly lower than the one reported for BanLec. If the same correlation as in BanLec exists between hemagglutination and mitogenic activity, the mitogenic activity of AcmJRL would potentially be very low.

Despite the absence of sequence identity between the β7β8 loops of these two proteins, two of the four amino acids facing the carbohydrate binding sites have conserved properties and orientations (Ala86 and Val88 in BanLec vs Val88 and Leu91 in AcmJRL), the two other amino acids being Tyr83 and His84 that contribute to the wall separating the two binding sites. Swanson *et al*.^69^ tested numerous mutations other than a threonine to replace His84, and found that His84Gly is the only one with a reduction of the mitogenic activity while preserving some antiviral potency. Interestingly, in AcmJRL, the residue equivalent to His84 in BanLec is precisely a glycine (Gly86), further suggesting a potential behavior of AcmJRL similar to the His84Thr BanLec mutant. However, two differences potentially preventing a high affinity of AcmJRL for HIV viruses remain, the absence of a residue equivalent to Tyr83 in AcmJRL, and the replacement of Gly14 by a glutamate in the carbohydrate binding site 1 of AcmJRL as discussed above. Therefore, further experimental data are required to assign a potential clinical use of AcmJRL as an antiviral agent against HIV, hepatitis C, and influenza viruses (and/or other viruses) while presenting a low mitogenic activity.

## Materials and Methods

### Hemagglutination activity and carbohydrate-inhibition assays

Sheep erythrocytes were from Biomérieux (France), rabbit erythrocytes were from Complement Technology, Inc. (USA) and rat erythrocytes from blood samples kindly provided by Profs. A.K. Cardozo and L. Ladrière from the ULB Diabetes Research Center (University of Brussels, Faculty of Medicine). Rat experimentation protocols were approved by the Animal Welfare and Ethical Committee of University of Brussels, Faculty of Medicine (CEBEA), protocol number 554N.

Hemagglutinating activity was performed in microtiter plates with V-bottom wells, using the two-fold serial dilution method, starting with a concentration of 1 mg/mL (65 µM) lectin in phosphate-buffered saline containing 150 mM NaCl. Rabbit, sheep and rat erythrocytes were washed six times with 150 mM NaCl and resuspended at a final concentration of 3% (v/v) in 150 mM NaCl. Twenty-five µL of lectin solution were mixed with 25 µL of 3% erythrocytes, serially diluted and incubated at room temperature and 37 °C for 1 h. Hemagglutination activity was also assayed in the presence of 50 mM of the divalent ions Mg^2+^, Mn^2+^ Zn^2+^ and Ca^2+^. In this case, Tris-HCl 50 mM buffer at pH 7.2 containing 150 mM NaCl was used to avoid precipitation of phosphate compounds. The hemagglutination activity was given as a titer (the reciprocal of the highest two-fold dilution) or expressed as the minimum protein concentration (μg/mL), which produced visible hemagglutination and denoted as Hemagglutinating Unit (HU). Specific activity was defined as the HU per milligram of protein. The carbohydrate-binding specificity was determined by the inhibition of hemagglutination of rat erythrocytes, that were only the hemagglutinated ones, with starting concentrations of 100 mM for D-mannose, methyl-α-D-mannopyranoside and methyl-α-D-glucopyranoside, 50 mM for D-glucose and N-acetyl-D-glucosamine, 15 mM for Manα1,3Man and Manα1,3(Manα1,6)Man and 10 mM for Manα1,3Manα1,6(Manα1,6)Manα1,3. The different saccharides were serially diluted from the starting concentrations. After addition of 25 µL of *A*. *comosus* lectin (187.5 µg/mL) to all microplate wells, 25 µL of each saccharide were mixed separately in the first microtiter plate well followed by two-fold serial dilution and pre-incubated at room temperature for 1 h. Then 25 µL of 3% mouse erythrocyte suspension were added to each well and the hemagglutination activity was evaluated after a further 1 h incubation period at room temperature and 37 °C. The lowest concentration of saccharides that visibly decreased agglutination was defined as the minimum inhibitory concentration (MIC).

### Chemical-induced unfolding transitions

Chemical-induced unfolding experiments were performed in 50 mM sodium phosphate buffer, pH 7.0. GdmCl solution was prepared in the same buffer, and the pH was adjusted to 7.0 with HCl or NaOH. Unfolding was studied at 20 °C. Samples at various GdmCl concentrations were allowed to equilibrate for at least 12 h (under these conditions, equilibrium is reached throughout the transition). Unfolding transitions were obtained by monitoring the changes in intrinsic fluorescence emission (λ_exc_ = 280 nm; λ_em_ = 370 nm) and circular dichroism (CD) at 222 nm, using a Tecan Infinite M200Pro fluorimeter and a Jasco J-810 spectropolarimeter, respectively, both thermostatically controlled. With all samples, the data were corrected for the contribution of the solution (buffer + denaturant). Denaturant concentrations in the samples were determined from refractive index measurements^70^ using a R5000 hand-held throughout. A protein concentration of 0.1 mg/mL (6.5 µM) was used for both intrinsic fluorescence and far-UV CD measurements.

Unfolding curves were analyzed on the basis of a two-state model for a homodimeric protein (N_2_ ⇌ 2U), according to equations derived by Bowie and Sauer^71^, and Gittelman and Matthews^72^. This analysis is based on the assumption that the free energy change for unfolding is linearly dependent on the denaturant concentration^73^ and it yields values of both the unfolding free energy in the absence of denaturant (i.e. ΔG°(H_2_O)_NU_) and m_NU_. The latter corresponds to the slope of the linear plots of the Gibbs free energy against denaturant concentration, i.e. δ(ΔG°)/δ[GdmCl], and thus reflects the sensitivity of the transition to the denaturant concentration. The midpoint of the transition, i.e. the denaturant concentration at which [N_2_] = [U], is given by C_m_ = ((R · T · lnC_Tot_) + ΔG°(H_2_O)_NU_)/m_NU_.

The program Grafit 5.0.10 (Erithacus Software Ltd.) was used for non-linear least-squares analysis of the data. Unless otherwise stated, errors are reported as standard deviations throughout.

### Isothermal titration calorimetry (ITC)

ITC titrations were performed on a MicroCal ITC200 (GE-Malvern) equipped with a 200 µL Hastelloy sample cell and an automated 40 µL glass syringe rotating at 1000 rpm. In order to avoid buffer mismatch and the generation of dilution heats, AcmJRL was first concentrated and exhaustively diafiltrated at 4 °C against Tris-HCl 50 mM, 150 mM NaCl, at pH 7.4 using 5000 MWCO Vivaspin 15R concentrator devices. Then, the diafiltration effluent was used to solubilize the saccharides. Control experiments indicated negligible heat signals for both buffer injections into AcmJRL and saccharide injection into buffer. In standard experiments, AcmJRL (0.5–0.8 mM) was titrated by an initial 0.4 µL injection followed by 19 injections (2 µL) of saccharides (10–50 mM) at an interval of 150 s.

The data so obtained were fitted via nonlinear least squares minimization method to determine the association constant (*K*_a_) and change in enthalpy of binding (*ΔH°*_*b*_) with a fixed binding stoichiometry (n = 1 and *n* = 2) for *n* equivalent binding sites, using ORIGIN 7 software v.7 (OriginLab). The Gibbs free energy of binging, *ΔG°*_*b*_, was calculated from *K*_a_ values and the entropic term, T*ΔS°*_*b*_, was derived from the Gibbs-Helmholtz equation using the recorded *ΔH°*_*b*_ values.

### Crystallization and soaking procedures

AcmJRL was concentrated to 17 mg/mL in a solution containing only water and crystallized at 20 °C using the hanging drop vapor diffusion method. The 4 μL hanging drops consisted of a 1:1 (v/v) mixture of the well solution and the protein solution. The crystal used to get the apo structure was obtained with a well solution composed of 15% (w/v) polyethylene glycol 3000 and 0.1 M sodium citrate buffer at pH 5.5. The crystal was transferred in a cryoprotectant solution containing 50% (v/v) 2-Methyl-2,4-pentanediol and 25% (w/v) polyethylene glycol 6000 before freezing in liquid nitrogen. For the AcmJRL:Man and AcmJRL: αMeMan complexes, the crystals were respectively obtained with well solutions of 4 M sodium nitrate in a 0.1 M Tris-HCl pH 8.5 buffer and 2 M ammonium citrate in a 0.1 M Bis-Tris propane buffer at pH 7.0. Crystal soakings were done in the original crystallization drops for 20 minutes before harvesting by adding 0.5 μL of 0.1 M D-mannose or 0.1 M methyl-α-D-mannopyranoside. In this case, the cryoprotectant solution contained 1.8 M ammonium sulfate and 45% (v/v) glycerol.

### Data collection, structures solution and determination

The diffraction data were collected on the Proxima1 beamline of the Soleil synchrotron. The data were indexed, integrated and scaled using XDS^74^. The crystal used for the apo AcmJRL structure belongs to the orthorhombic space group P2_1_2_1_2_1_, and the crystals of the two complexes to two different hexagonal P6_4_22 space group. The structure of AcmJRL was solved by molecular replacement with Phaser^75^ using the structure of the banana lectin as search model (pdb code 1 × 1 V)^31^. The refinement and model building cycles were respectively performed with phenix.refine^76^ and Coot^77^. A summary of the relevant statistics of the data collection and refinement is given in Table 3. Protein superimposition was performed using the Secondary Structure Matching algorithm implemented in Coot. The figures were prepared using PyMOL (The PyMOL Molecular Graphics System, Version 1.7.4.3 Enhanced for Mac OS X, Schrödinger, LLC.).

The coordinates and structure factors of the apo AcmJRL, AcmJRL:Man and AcmJRL:MeαMan structures have been deposited in the Protein Data Bank with the PDB ID codes 6FLW, 6FLY and 6FLZ, respectively.

Additional information can be found in the Supplementary Materials and Methods section.

### Data availability

The data that support the findings of this study are available from the authors upon reasonable request.

## Electronic supplementary material

Supplementary information
