# Supplementary material for: Biochemical and structural characterization of a mannose binding jacalin-related lectin with two-sugar binding sites from pineapple (Ananas comosus) stem
Source: Sci Rep. 2018 Jul 31;8:11508. doi: 10.1038/s41598-018-29439-x (PMC6068142; doi:10.1038/s41598-018-29439-x)
Supplement: Supplementary file 1 — Supplementary information [file 41598_2018_29439_MOESM1_ESM.pdf]

Biochemical and structural characterization of a mannose binding jacalin-related lectin with two-sugar binding sites from pineapple (*Ananas comosus*) stem

Mohamed Azarkan[1], Georges Feller[2], Julie Vandenameele[3], Raphaël Herman[4], Rachida El Mahyaoui[1], Eric Sauvage[4], Arnaud Vanden Broeck[4], André Matagne[3], Paulette Charlier[4], Frédéric Kerff[4]

[1] Université Libre de Bruxelles, Faculty of Medicine, Protein Chemistry Unit, Campus Erasme (CP 609), 808 route de Lennik, 1070 Brussels, Belgium.

[2] Laboratory of Biochemistry, Center for Protein Engineering-InBioS, Institute of Chemistry B6a, University of Liège, 4000 Liège, Belgium.

[3] Laboratory of Enzymology and Protein Folding, Centre for Protein Engineering-InBioS, Institut de Chimie B6, University of Liège, 4000 Liège, Belgium.

[4] Laboratory of crystallography, Center for Protein Engineering-InBioS, B5a, University of Liège, 4000 Liège, Belgium.

Supplementary materials and methods

Chemicals

Stem bromelain (lot number 80K1406), S-methylmethanethiolsulfonate (MMTS), Z-Phe-Arg-AMC, Z-Arg-Arg-AMC and Boc-Gln-Ala-Arg-AMC, TPCK-treated bovine trypsin, D-glucose, D-mannose, Methyl-α-D-mannopyranoside, methyl-α-D-glucopyranoside, *N*-acetylglucosamine (GlcNAc) and ultrapure guanidinium chloride (GdmCl) were purchased from Sigma-Aldrich (Steinhein, Germany). D-mannose was from Fluka (Buchs, Switzerland), Manα1,3Man, mannotriose (Manα1,3(Manα1,6)Man) and mannopentaose (αMan(1,3)(αMan[1,6])-αMan(1,6)(αMan[1,3])Man) were provided by Dextra Laboratories (United Kingdom). Precast gels (ExcelGels, gradient 8-18% and Ampholine PAGplate gels) and silver staining kit were from Amersham Biosciences, part of GE Healthcare (Uppsala, Sweden). Molecular mass standards and compatible mass spectroscopy protein sequencing silver staining kit were purchased from Bio-Rad Laboratories (Hercules California, USA). All the other chemicals were of analytical grade.

Sample preparation

Stem bromelain powder (10.0 g) was set in sodium acetate buffer 100 mM at pH 5.0 containing 1mM EDTA and 20 mM methylmethanethiolsulfonate (MMTS) under gentle stirring for 45 min at room temperature. MMTS was used to block selectively and reversibly the cysteine proteases present in stem bromelain sample to prevent proteolytic degradation of lectin. The resulting suspension was then subjected to centrifugation (40,000 x g, 4°C, 30 min). The insoluble material was discarded and the supernatant constituted the starting material. Enzymatic activity measurements, both against fluorogenic (Z-Phe-Arg-AMC and Z-Arg-Arg-AMC) and chromogenic (DL-BAPNA and Boc-Ala-Ala-Gly-pNA) substrates, demonstrated that no residual amidase activity could be detected, demonstrating that the cysteine protease activity was quantitatively blocked by MMTS.

Lectin purification

The whole stem bromelain soluble protein fraction was dialyzed against Tris-HCl 50 mM buffer pH 7.2 containing 500 mM NaCl before to be loaded onto a mannose-Agarose column pre-equilibrated with the dialysis buffer. After washing with the same buffer, AcmJRL was eluted with the same buffer containing 1 M D-mannose. The eluted fractions were pooled and extensively dialyzed first against water at 4 °C and then against Tris-HCl 50 mM buffer pH 7.2 containing 150 mM NaCl and concentrated before to be submitted to FPLC-gel filtration chromatography.

To further improve the AcmJRL purity and homogeneity, the sample eluted from D-mannose-Agarose was submitted to FPLC-Superdex 75 pg, on an AKTAprime system. The column (Hiload 16/600) was equilibrated and eluted with a 50 mM Tris–HCl buffer at pH 7.4 containing 150 mM NaCl and 100 mM mannose to prevent interactions between the lectin and the gel matrix. Running conditions were 1.0 mL/min, 3.0 mL/fraction and 0.6 kPa. All the solutions were degazed and filtered through 0.22 µm before use.

Assessment of oligomerization status of AcmJRL in solution using FPLC-gel filtration chromatography

Molecular size of AcmJRL was measured using the FPLC-Superdex 75 pg column following the same experimental conditions described in the previous paragraph. The column was calibrated with the commercially available (Aldrich-Sigma) protein molecular weight markers cytochrome C from horse heart (12.4 kDa), carbonic anhydrase from bovine erythrocytes (29.0 kDa) and the home-purified proteins hen egg white lysozyme (14.4 kDa),

ficins A (23.8 kDa) and D1 (24.5 kDa) from *Ficus carica* latex[1] and the minor chitinase (26.5 kDa) from *Carica papaya* latex[2]. The calibration curve was established by plotting molecular weight versus V_e/V_0, where V_e is the volume of effluent collected from the point of sample application to the center of the effluent peak and V_0 is the column void volume determined by using blue dextran (2,000 kDa).

<u>Accurate molecular mass determination by ESI-Q-TOF-MS</u>

The analysis was performed on an ESI-Q-TOF (Waters, Micromass) in positive ion mode. The samples were analyzed by ESI-Q-TOF-MS at a protein concentration of 10 µM, 30% ACN, 0.5% formic acid (final) in ammonium acetate 25 mM. Peak intensity is communicated under the mass to charge ratio (*m/z*) value on spectra. Calibration was performed using clusters of phosphoric acid in *m/z* range 90 to 3000, corresponding to raw spectra acquisition range. Resolution obtained was 8600. Mass accuracy of the instrument is 100 ppm. Spectra deconvolution technique of calculation is the maximum entropy: *max ent1*.

<u>N-terminal sequence determination</u>

Protein samples were concentrated on PVDF membranes before being submitted to N-terminal amino-acid micro-sequencing, using gas-phase Edman degradation in an Applied Biosystems 476A protein sequencer (Foster City, CA).

<u>Glycan detection</u>

Glycoprotein detection was performed on SDS–PAGE using the Pierce Glycoprotein Staining Kit (Thermo scientific) according to the manufacturer's instructions. Briefly, gels were fixed, washed and oxidized with periodic acid. After thoroughly washing to remove residual periodic acid, the gels were stained, reduced and carefully washed. Glycoproteins appeared as magenta bands. Horseradish peroxidase and soybean trypsin inhibitor were used as positive and negative controls, respectively.

<u>SDS-PAGE and PAGE experiments</u>

The SDS-PAGE experiments were performed on precast gels (ExcelGel, 245x110x0.5 mm, gradient 8-18%) using the Multiphore II kit from Amersham Biosciences, running conditions 600 V, 50 mA and 35 W at constant temperature (15.0±0.1)°C or on slab gels using the Mini-protean III cell from Bio-Rad Laboratories at 4.0°C. The resolving gels (pH 8.8, 12.0% acrylamide) were run at a constant current (35 mA). The stacking gel consisted of 4.0% polyacrylamide, pH 6.8. The upper and lower chambers contained Tris/glycine, pH 8.3, with 0.1% SDS. The separation was towards the anode and bromophenol blue was used as the tracking dye. Staining was made with Coomassie Brilliant Blue R-250 or silver staining procedures. Molecular weight standards were hen egg white lysozyme (14.4 kDa), soybean trypsin inhibitor (21.5 kDa), carbonic anhydrase (31.0 kDa), hen egg white ovalbumin (45.0 kDa), bovine serum albumin (66.2 kDa) and rabbit muscle phosphorylase b (97.4 kDa). Native PAGE experiments were carried out on slab gels using a Mini Protean III system (Bio-Rad Laboratories). The separating gels were 7.5% acrylamide, pH 4.3, and the stacking gels were 3.0%, pH 6.8. The buffer in both the upper and lower electrode chambers was β-alanine/acetic acid, pH 4.3. The separation was towards the cathode and methylene green was used as tracking dye. Electrophoresis was carried out at 4°C by applying a current of 40 mA/slab gel for 75 min.

<u>Protein determination</u>

Protein concentrations were determined spectrophotometrically using $\varepsilon_{280\ nm}$ = 19940 M^{-1} cm^{-1}. This value was calculated using information from the SwissProt Data Bank (MW, Trp and Tyr

chromophores content and S—S bonds) and the known ε values of the various chromophores[3]. Absorbances were measured with a Varian UV-Visible Cary 50 spectrophotometer.

References

1. Azarkan, M. *et al.* Selective and reversible thiol-pegylation, an effective approach for purification and characterization of five fully active ficin (iso)forms from *Ficus carica* latex. *Phytochemistry* **72,** 1718–1731 (2011).

2. Huet, J. *et al.* Structural characterization of two papaya chitinases, a family GH19 of glycosyl hydrolases. *Cell. Mol. Life Sci.* **63,** 3042–3054 (2006).

3. Gill, S. C. & von Hippel, P. H. Calculation of protein extinction coefficients from amino acid sequence data. *Anal. Biochem.* **182,** 319–326 (1989).

Supplementary Figures

a

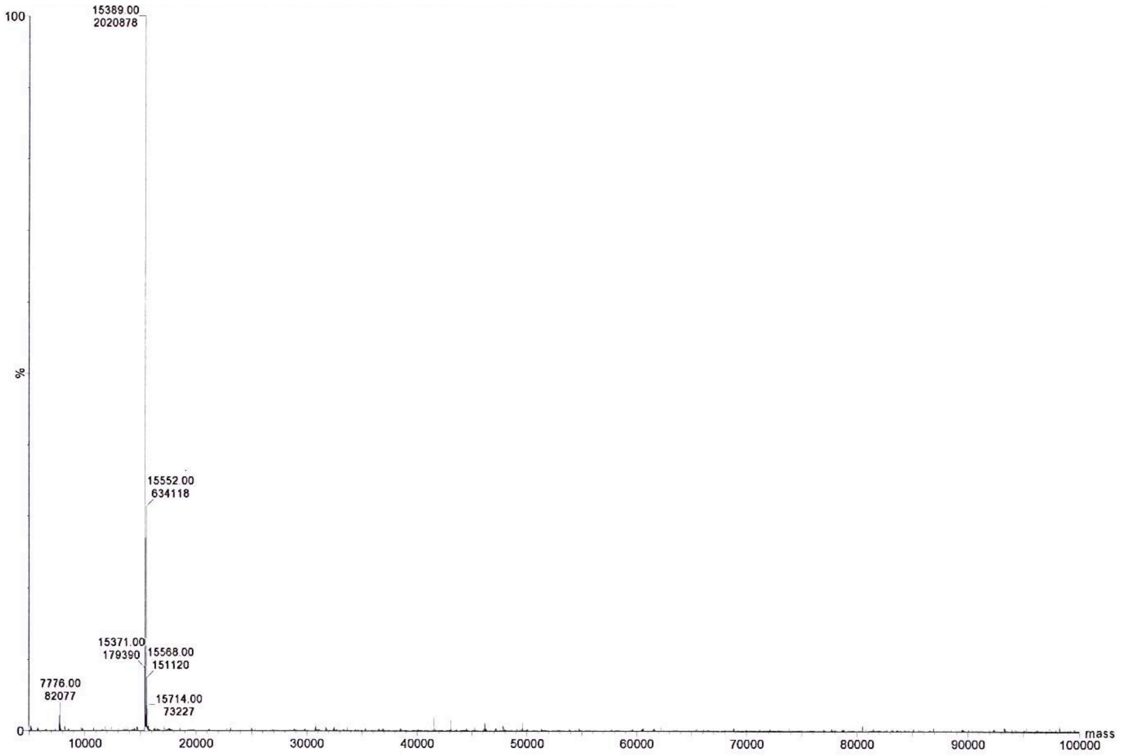

b

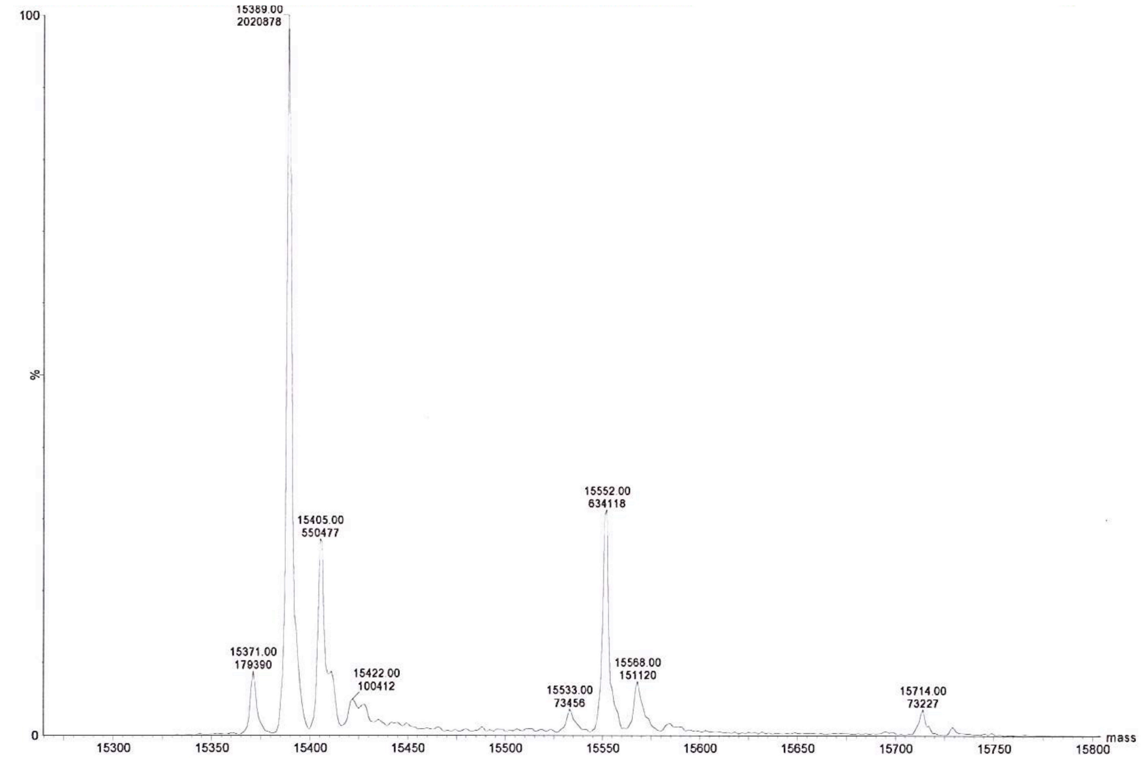

Supplementary Figure 1. (**a**) Maxent1 deconvolution of the ESI-Q-TOF mass spectrum of the purified AcmJRL protein sample. (**b**) Zoom of (**a**) around the peak of interest.

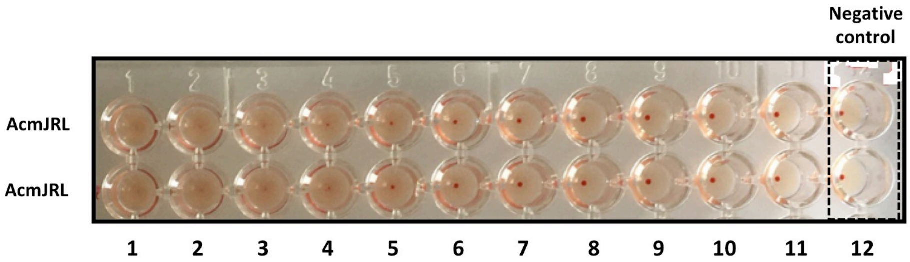

Supplementary Figure 2. Hemagglutination of rat erythrocytes by AcmJRL.
AcmJRL final concentrations are 0.5, 0.25, 0.125, 0.0625, 0.0313, 0.0156, 0.0078, 0.0039, 0.0019, 0.0098 and 0.0005 mg/mL from well 1 to well 11, respectively. Well 12 is the negative control.

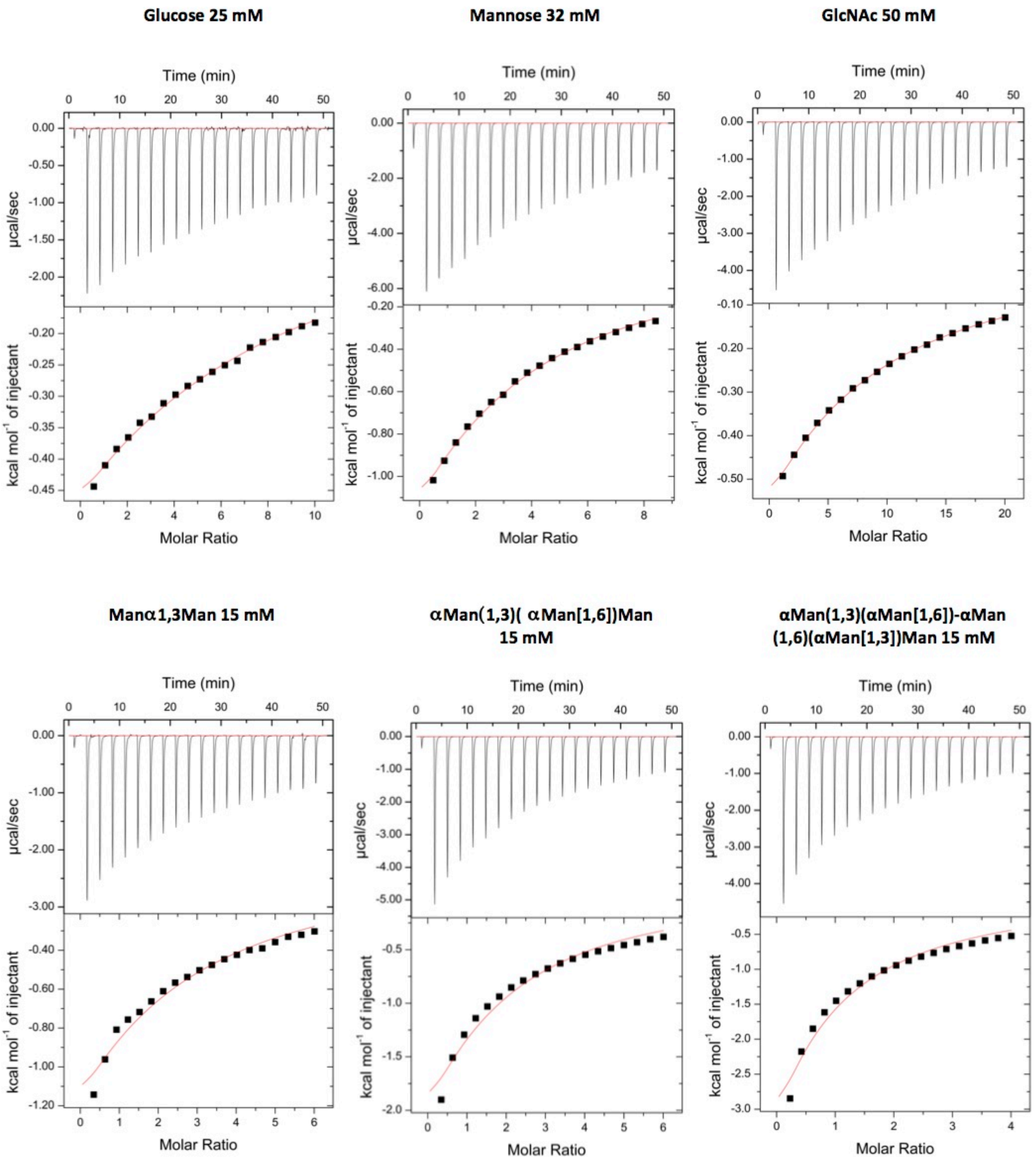

Supplementary Figure 3. Isothermal titration calorimetry of saccharide binding to AcmJRL at 25 °C. Upper panels: exothermic microcalorimetric traces of saccharide injections (at the indicated concentrations) into AcmJRL solution (525 µM or 800 µM for mannose). Lower panels: Wiseman plot of heat releases versus molar ratio of injectant/protein in the cell and nonlinear fit of the binding isotherm for 2 equivalent binding sites.

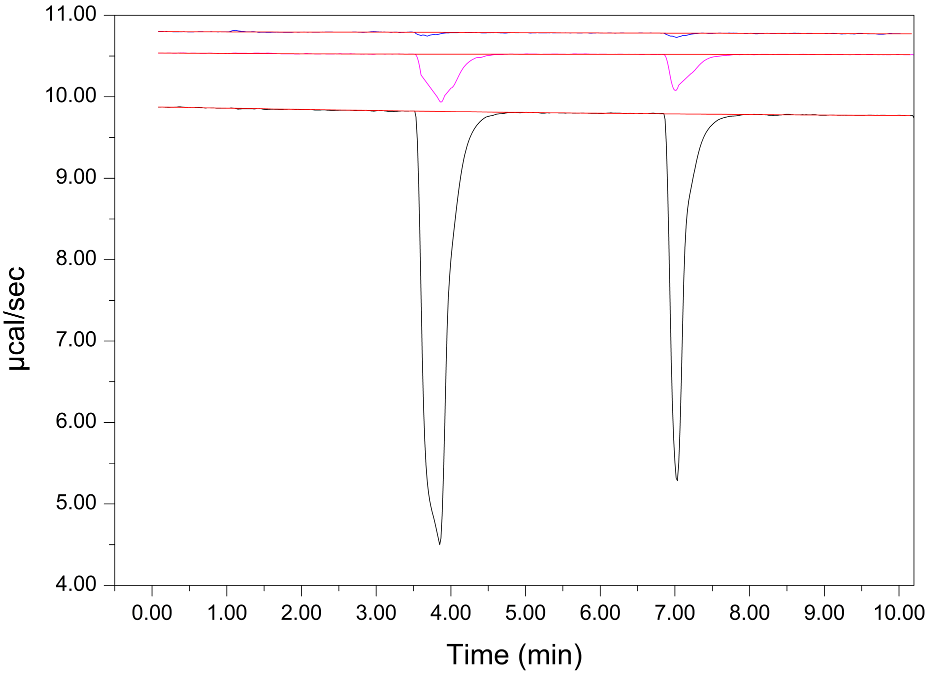

Supplementary Figure 4. Experimental control of the binding enthalpy of mannose to AcmJRL. Injections of 10 µl (first injection) and 5 µl (second injection) of AcmJRL 0.8 mM into mannose 150 mM (lower black thermogram), of buffer into mannose 150 mM (middle magenta thermogram) and of AcmJRL 0.8 mM into buffer (upper blue thermogram). Both latter thermograms have been shifted upward for clarity. Red baselines delineate the peak area for integration. Subtraction of the blank contributions (magenta and blue) from the total heat of binding (black) allows to evaluate the binding enthalpy of mannose to 45 kJ mol-1 or 22.5 kJ mol-1 for two equivalent binding sites, in good agreement with the value of 43.2 kJ mol-1 (n=1) or 20.0 kJ mol-1 (n=2) derived from the fit results (Table 2 and Supplementary Table 2).

Supplementary Tables

Supplementary Table 1. Inhibition of rat erythrocytes hemagglutination by AcmJRL

Saccharide	MIC (mM)
D-Glucose	3.12
GlcNAc	3.12
D-Mannose	6.25
Methyl-α-D-mannopyranoside	1.56
Methyl-α-D-glucopyranoside	1.56
Manα1,3Man	0.94
Mannotriose[(1)]	0.47
Mannopentaose[(2)]	0.32

[(1)] αMan(1,3)(αMan(1,6))Man

[(2)] αMan(1,3)(αMan(1,6))-αMan(1,6)(αMan(1,3))Man

Supplementary Table 2. Thermodynamic parameters of saccharide binding to AcmJRL at 25 °C for one binding site (*n*=1).

**Saccharide**	K_a **M^{-1}**	ΔG°_b **kJ mol^{-1}**	ΔH°_b **kJ mol^{-1}**	TΔS°_b **kJ mol^{-1}**
D-Glucose	76 ± 2	-10.7 ± 0.3	-48.7 ± 1.1	-38.0
GlcNAc	82 ± 1	-10.9 ± 0.1	-52.7 ± 0.3	-41.8
D-Mannose	145 ± 2	-12.3 ± 0.2	-43.2 ± 0.4	-30.8
Manα1,3Man	318 ± 27	-14.3 ± 1.2	-32.3 ± 1.7	-18.0
Mannotriose[(1)]	513 ± 45	-15.5 ± 1.3	-36.3 ± 1.8	-20.8
Mannopentaose[(2)]	1040 ± 143	-17.2 ± 2.4	-31.0 ± 2.1	-13.8

[(1)] αMan(1,3)(αMan(1,6))Man

[(2)] αMan(1,3)(αMan(1,6))-αMan(1,6)(αMan(1,3))Man
